# College of Medical Practitioners, St. Louis

**Published:** 1883-01-20

**Authors:** 


### *COLLEGE OF MEDICAL PRACTITIONERS, ST. LOUIS.*

This, as appears from a circular received, is not so much a College as an Assocation of Medical Practitioners composed—

First. Of Faculty members, limited to twelve.

Second. Honorary members, unlimited.

Third. Associate members, unlimited.

The College or Association is open to the whole medical profession, graduates and non-graduates, and certificates of attendance will be given without an examination.

